# Assessing effectiveness and skill transferability in multi-platform simulated training for robotic surgical skills: a systematic review

**DOI:** 10.1007/s11701-025-03090-x

**Published:** 2025-12-29

**Authors:** Wai Miu Emma Liu, Katy-Anne Burr, Charvi Dave, Callum Pearse, Yang Li, Julien Quarez, Alejandro Granados, Ben Challacombe, Prokar Dasgupta, Nicholas Raison

**Affiliations:** 1 GKT School of Medical Education, King’s College London, London, UK; 2 School of Biomedical Engineering & Imaging Sciences, King’s College London, London, UK; 3 Department of Urology, Guy’s and St Thomas’ NHS Foundation Trust, London, UK; 4 Department of Urology, King’s College Hospital, London, UK

**Keywords:** Robotic surgery, Surgical education, Skill transfer, Surgical simulation, Simulated training

## Abstract

With more robotic platforms approved for clinical use, interest in assessing skill transferability has increased. Skill transfer has yet to be studied amongst trainees in early stages of robotic training. This systematic review evaluated skill transferability of robotic platforms amongst trainees in simulated settings. The systematic review was conducted according to PRISMA guidelines. Five databases were searched from inception until 20 January 2025. Inclusion criteria had studies using simulation-based modalities, more than one robotic platform, and objective metrics or global rating scales for skills assessment. Search identified 609 unique studies. Five studies were included. Platforms analysed were Da Vinci X, Si and single-port system, Hugo RAS and CMR Versius. Consistent performances were seen across platforms amongst novices and experts, whilst intermediates exhibited reduced scores (GEARS − 2.9). Prior robotic surgical experience was the main driver for better baseline performance and skills progression. Skill transferability was demonstrated in cross-platform simulated training amongst experts and novices, indicating that core robotic surgical skills can be applied across systems. Less evidence of skill transfer in intermediate-level participants suggests that those still consolidating their technical proficiency may be more sensitive to platform-specific differences. Console design variations appeared to influence transferability. Incorporating cross-platform training could enhance trainees’ understanding of the fundamental principles of robotic surgery, equipping them with more adaptable skillsets suited to various platforms. This has important implications for surgical training, as during the trainees’ learning phase, they should be aware of the potential decline in technical performance when transitioning between platforms.

**Supplementary Information:**

The online version contains supplementary material available at 10.1007/s11701-025-03090-x.

## Introduction

The Da Vinci Surgical System (Intuitive Surgical, Sunnyvale, CA, USA) has long dominated the market since its first FDA approval in 2000. However, in the last 15 years, the number of surgical robotic companies has risen, with a growing diversity in robotic platforms available for surgical use [1–4]. With the increased prevalence of surgical robotic systems in the operating room, there is an increasing drive to establish a curriculum that can most effectively train surgical trainees.

Numerous training curricula have been proposed, utilising various training modalities and assessment methods [5, 6]. Curricula tend to use a combination of training methods with the growing incorporation of deliberate practice, whereby validated benchmarks should be reached before advancing to more complex tasks or another modality [7–9]. Virtual reality (VR) training generally used initially to allow familiarisation of instruments and proficiency with basic robotic skills. It is also a preferred method for early training due to its convenience, cost-effectiveness and safety [10]. VR training permits trainees to familiarise themselves with the controls, user interface and cameras of the platform. Furthermore, abstract tasks on the simulator tasks allow for the acquisition of core technical skills including depth perception, bimanual dexterity, tissue handling, Endowrist manipulation, camera and clutch control [8, 11]. After competency is achieved in these domains, trainees advance to more technical basic skills, such as suturing, knot-tying and energy device application [12]. These can be done through VR simulation, dry lab or wet lab models [13, 14]. Dry lab and wet lab models are incorporated more and preferred in later stages of training as they offer a higher fidelity [15].

Heterogeneity is also seen in the assessment of robotic skills. Global rating scales, such as the Global Evaluative Assessment of Robotic Surgery (GEARS) [16], are popular to measure robotic surgical proficiency. However, the reliance on an trained assessor to conduct the rating means this method is unscalable and can introduce bias [17]. Objective metrics, such as hand kinematic data captured by robotic systems, offer greater accuracy and resource efficiency; however, access to these data remains tightly controlled by device companies, limiting their practical application [18, 19]. The use of multiple robotic platforms during training has also increased over the past few years [20–22]. However, they mostly have been incorporated in an introductory capacity, providing trainees with brief exposure rather than structured training.

Modern robotic surgical platforms typically consist of bedside instruments that are controlled by a surgeon console at a distance. Surgeon and patient consoles can vary greatly across different robotic platforms. This heterogeneity presents potential challenges regarding skill transferability between platforms. Current studies have focussed on skill transfer between platforms amongst expert surgeons [23, 24], leaving a gap in the understanding of skill transferability during early stages of training. Assessing the value of incorporating cross-platform training in early robotic training can provide important insights in the optimisation of robotic curricula.

The aim of this systematic review is to evaluate the effect of using multi-platform simulation basic robotic skills, and to assess the transferability of skills across different robotic operating platforms in early robotic training.

## Methods

The Preferred Reporting Items for Systematic Reviews and Meta-Analyses (PRISMA) guidelines were followed when conducting this systematic review [25]. A systematic review protocol was developed a priori to guide the search strategy, inclusion criteria and data synthesis. The protocol was not registered in a public registry but is available from the corresponding author upon request.

### Search strategy

A comprehensive search of five databases was conducted; PubMed, Medline, Embase, Scopus and Clinicaltrials.gov. The search strategy included MeSH terms related to modalities of robotic training, such as “dry lab” and “virtual simulation”, and the names of varying robotic surgical systems. Search syntax was done in a stepwise format to ensure capture of studies that included at least two robotic systems. The full list of search terms, the formatting of search terms for each database and the number of journals yielded from each search can be viewed in the supplementary information.

To ensure comprehensive coverage of literature, supplementation of citation searching was included. Forward citation searching was performed using PubMed to identify newer articles that had cited our included studies. Backward citation searches involved manually screening the references of all included articles. Any identified studies through this method were subjected to the same citation searching process.

## Study selection and data extraction

Studies included were all published in peer-reviewed scientific journals. Conference abstracts were excluded from analysis. The Population, Intervention, Comparison, Outcome framework was used to formulate our inclusion and exclusion criteria, see Table 1 [26].

**Table 1:** Eligibility criteria for systematic review

Included	Excluded
Individuals with none or limited experience in robotic surgery, such as students or surgical trainees	Robotic procedures conducted on patients
Simulation-based training modality, including VR simulation, dry lab models, cadaveric models, or phantom models	
Performance data comparing ≥ 2 robotic platforms OR performance data of one platform with the individual’s experience on another collected	Data on only one robotic platform
Outcomes assessed with objective performance metrics, global rating scale, or validated subjective assessments	Studies only providing opinion-based evaluations
RCTs, cohort studies, observational studies	Case reports, Single-surgeon studies, grey literature

RCTs = randomised controlled trials, VR = virtual-reality

The studies retrieved from the search were deduplicated. Title and abstract screening and subsequent full text screening were done blinded by two reviewers (W.L. and K.B.). Data collected from each study was recorded on a table on Microsoft Excel. Data collected included details on study population, study design, assessment methods and outcomes. Study population consisted of the country of study, number of participants, the participants’ specialty, level of training and details on how competency levels were defined. Study design included the primary aim of the study, types of robotic platforms used, and the types of training modalities used. Assessment methods and outcomes included details on the tasks set out for each participant, task endpoint definition, methods used to measure outcome and their results. Skill transferability was defined as the maintenance or improvement of performance metrics when a user transitions from a robotic platform to a different one.

## Risk of bias assessment

The Risk Of Bias In Non-randomised Studies – Of Interventions, Version 2 (ROBINS-I V2) was used for quality assessment for each included study after full-text screening. This was done blinded by two reviewers (W.L. and K.B.). The *robvis* tool was used to create the ‘traffic light’ plots and the weighted bar plots to visualise the risk of bias assessments completed [27].

### Data analysis

Due to the heterogeneity of study methodology and reporting of outcomes, data was analysed with a narrative synthesis approach. This involved the summarising and synthesising of the results in accordance to the PRISMA guidelines and the Synthesis without meta-analysis (SWiM) guidelines [25, 28].

## Results

**Fig. 1:**
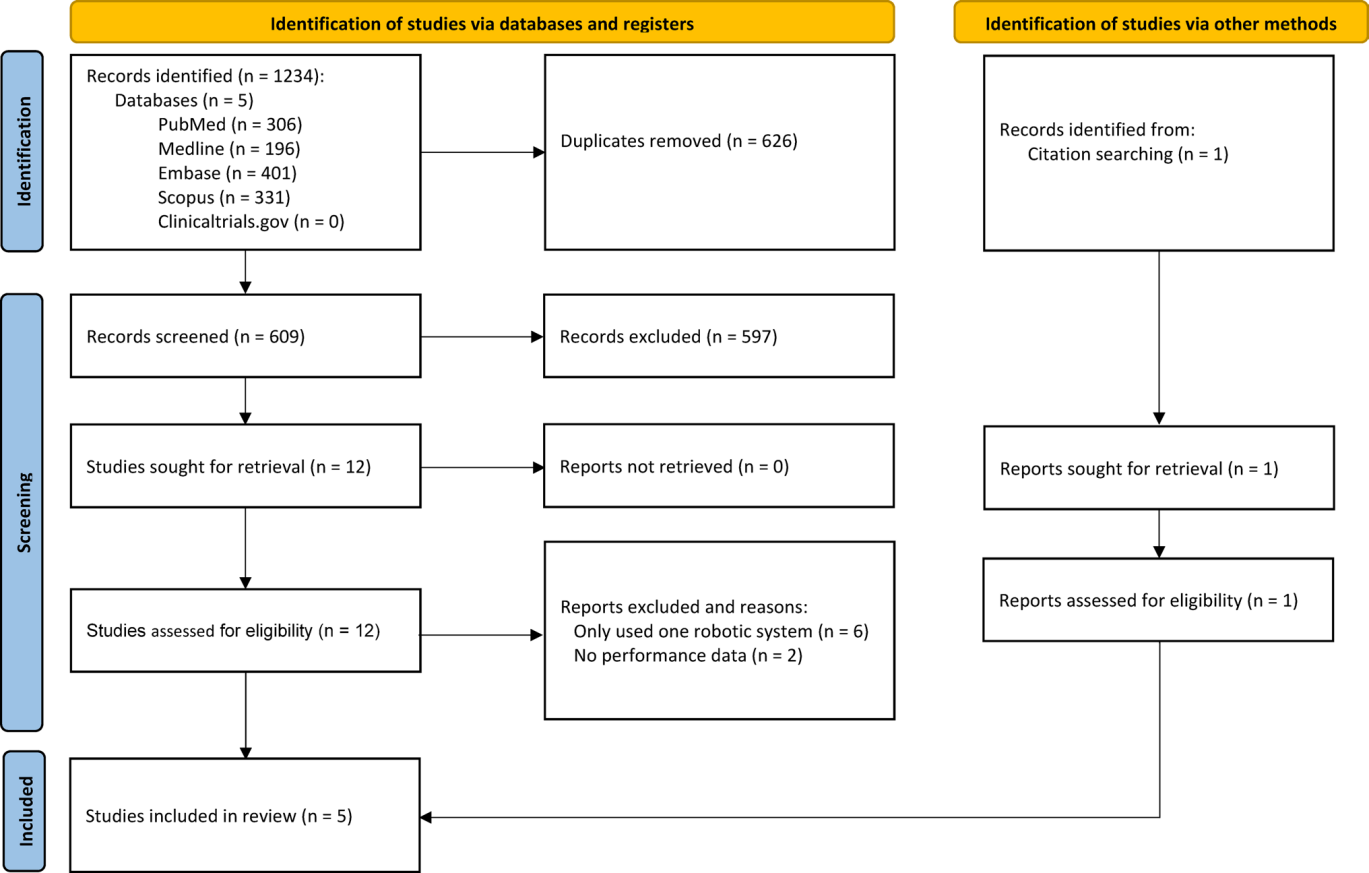
PRISMA 2020 flow diagram showing the literature search and selection process [25]

The search strategy yielded 609 unique studies from the mentioned databases. These studies underwent title-abstract screening following the aforementioned eligibility criteria. Twelve studies were identified to be eligible for full-text screening. One study was found through citation searching. A total of 5 studies were included for analysis of this study (Fig. 1). Characteristics and findings of included studies are displayed in Table 2. The heterogeneity in study methodology and outcome measures limited the ability to conduct a meta-analysis, so the results were narratively synthesised.

**Table 2:** Summary of characteristics and outcomes of the included studies

Author, Year	Country	Primary aim	Study Population		Intervention			Assessment methods and Outcomes				
			Population size and characteristics	Subgroup size and Criteria	Robotic Platforms Included	Description of repetitions/training flow	Endpoint definition	Objective metrics	Global rating scales	Outcome	Participant satisfaction	Outcome
Butterworth et al. 2021 [20]	USA	Surgical training using VR simulator and cadavers on the Versius amongst surgeons of varying Da Vinci experience	17 gynaecology, general, colorectal, urology trainee surgeons	3 groups: 1. <5 robot procedures (*n* = 4) 2. 5 - <30 robotic procedures (*n* = 8) 3. ≥30 robotic procedures (*n* = 5)	Versius Surgical System simulator	15 tasks on the Versius Surgical simulator and specialty-specific tasks with cadaveric specimens on the Versius were each done 4 times over a 4- day training programme	Task completion	(simulator) - task completion time - combined instrument path length - combined instrument angular path	(wet lab) GEARS	Objective metrics from the simulation broadly aligned with global rating scale scores from the wet lab exercises across competency groups Greatest GEARS increase in wet lab tasks in those most experienced (+ 4.3) compared to those with some (+ 1.5) and no (+ 2.7) robotic experience	NA	NA
Ghazi et al. 2023 [29]	USA	Assess skill transferability between the Da Vinci MP to SP platforms using a phantom model in three competency groups	15 urology trainees	3 groups: 1. Novice (*n* = 5): limited MP cases (< 10); no prior SP experience 2. Intermediate (*n* = 5): >5 years of Da Vinci MP experience and > 250 cases of robotic MP cases and no SP experience 3. Expert (*n* = 5): > 250 cases in MP and > 50 cases of SP	Da Vinci SP system Da Vinci Xi MP system	Task involved 3D printed phantom models of urethrovesical anastomosis in robot-assisted radical prostatectomy using the MP robot, then repeated the same task within 2 weeks using the SP robot	Task completion	NA	GEARS RACE	Novices maintained low GEARS and RACE scores from MP to SP novices (GEARS: 17.3 to 18.1, RACE: 15.9 to 16.3) Significant score reduction seen with partial skill transfer in intermediate group (GEARS: 27 to 24.1, −2.9; RACE: 26.9 to 21.8, −5.1)	SURG-TLX Difficulty scoring for 3 domains - camera movement - EndoWrist movement - avoiding instrument collisions	Higher SURG-TLX and difficulty scores for SP
Larkins et al. 2022 [21]	Australia	Transferability of basic console operating between Hugo™ RAS and Da Vinci skills in a simulated setting	10 trainees from general surgery and urology	Two groups of 5 with equivalent spread of robotic experience	Hugo™ RAS dV-Trainer^®^	simulation exercises via Mimic^®^ Simulation Portal - Pick and place - Pegboard 1 - Matchboard 1 - Thread the rings 2 Each group performed tasks on either system, then crossed over afterwards Study kept platforms blinded, labelling them console A and console B	Task completion and the pre-defined outcome metrics was met on two consecutive attempts	- Task Completion time - Economy of motion - Workspace range - Instruments out of view - Excessive force - Instrument drops and collisions	NA	No significant improvement with cross-platform training Non-significant faster completion times for group that started with console B	Participant Satisfaction questionnaire	50% stated skills were ‘absolutely transferable’ and 50% stated skills were ‘somewhat transferable’
Reitano et al. 2023 [30]	France	Evaluate basic robotic skills of novices with a medical and non-medical background with dry lab models	29 medical and non-medical students (engineers)	2 groups: 1. Medical Students (*n* = 13) 2. Non-medical Students (*n* = 14)	Da Vinci X DaVinci Si Versius Surgical System Hugo™ RAS	Task involved a dry lab model in pelvic trainer where 6 pins are moved from non-dominant to dominant controller then to a colour matching target Same task performed on each robotic system in no specific order	maximum of 180 s to complete task	Bimanual coordination task metrics - time taken - number of pins moved - number of task completion	GEARS	No difference in performance between subgroups All tasks completed under allotted time on the Da Vinci X No task completion on CMR Versius	NA	NA
Sighinolfi et al. 2023 [31]	Italy	Evaluate performance of trainees of varying (da Vinci) robotic exposure during first experience with the Hugo RAS simulator	71 medical students and surgical trainees	3 groups: 1. naïve (*n* = 55): 2. laparoscopic surgeons (*n* = 6) 3. robotic surgeons (*n* = 10)	Hugo™ RAS	Simulator ‘pick and place’ exercise from Mimic^®^ Technologies on the Hugo™ RAS Task performed twice, with assessment on the second time	Task completion	- Time to exercise completion - Economy of motion - Master workspace range - Excessive force - Time of instruments out of view - Number of collisions	NA	Significantly faster task completion times in robotic surgeons than laparoscopic surgeons (*p* = 0.004) and naïve participants (*p* = 0.002) Previous robotic experience is the main driver for skill transferability	NA	NA

GEARS = Global Evaluative Assessment of Robotic Skills, RACE = Robotic Anastomosis Competency Evaluation, SURG-TLX = Surgical Task Load Index, SP = single port, MP = multiport

### Study characteristics

All studies included were observational studies. Study populations ranged from 10 to 71 participants and their experience levels varied. Two studies included medical or undergraduate students [30, 31]. Three studies utilised their own criteria to form three competency subgroups. Butterworth et al. formed the competency subgroups by robotic caseload, Ghazi et al. utilised a combination of years and robotic caseload, and Sighinolfi et al. formed subgroups based on years of laparoscopic and robotic experience [20, 21, 31].

#### Robotic platforms

All studies included at least one Da Vinci multiport platform [20, 29–31], or its simulator equivalent, the dV-trainer [21]. Three studies directly compared surgical systems: one between the Hugo RAS and the dV-trainer [21], one between the Da Vinci Xi MP system and the Da Vinci SP system [29], and one between the Da Vinci X, the Da Vinci Si, the Versius system and the Hugo RAS [30]. Two studies utilised a single-robot approach, either the Hugo RAS [31] or the Versius [20], and correlated performance data with the participant’s prior experience on the Da Vinci platform.

## Training modalities

Two studies used exclusively VR simulation tasks, both requiring participants to complete each task [21, 31]. Two studies utilised only dry lab models [29, 30]. Butterworth et al. used both VR simulation and cadaveric specimens with differing tasks [20].

## Assessment methods

Assessment methods used for each study varied due to the range of training modalities.

Objective metrics were used in four studies [20, 21, 30, 31]. Three studies exclusively used VR simulation and the metrics that can be broadly divided into three categories: task completion time, quality and efficiency metrics, and risk and safety metrics (20,). Task-specific objective metrics were used for the remaining study [30].

Three studies using wet-lab or dry-lab models applied global rating scales [20, 29, 30]. Two used GEARS [20, 30], whilst one utilised both GEARS and the Robotic Anastomosis Competency Evaluation (RACE) [29].

Two studies also collected the participants’ opinion using different robotic platforms in the study [21, 29]. This included the participants’ perceptions regarding the value of multiplatform exposure [21], the use of the Surgical Task Load Index (SURG-TLX) and a difficult rating on three domains: camera movement, EndoWrist movement and avoiding instrument collisions [29].

### Platform-specific skill transfer findings

Three studies directly compared the performance data of the same task between robotic platforms [21, 29, 30].

Ghazi et al. reported stable GEARS and RACE scores amongst the novice participants (GEARS: 17.3 to 18.1; RACE: 15.9 to 16.3) and the expert participants (GEARS: 27 to 25; RACE: 25.7 to 23.7) when transferring from the Da Vinci MP to SP system. However, significant reduction was reported for the intermediate subgroup (GEARS: 27.0 to 24.1, −2.9; RACE: 26.9 to 21.8, −5.1) [29]. Intermediate participants scored similarly for both platforms in specific subdomains in GEARS and RACE, suggesting that a few subdomains demonstrated skill transfer between these platforms.

Larkins et al. reported no significant differences in the time to proficiency or to pass when transferring between the Hugo RAS and the Da Vinci. The study author reported faster times were recorded in 3 out of the 4 tasks in the subgroup that used console B, suggesting one system being more intuitive than the other. However, due to de-identification of the platforms and the lack of significant differences in performance between the systems, the ability to draw platform-specific conclusions has been limited [21].

Reitano et al. compared 4 platforms: the Da Vinci X, Da Vinci Si, Versius system, and the Hugo RAS, but statistical analysis were done between subgroups and not between surgical platforms [30]. There was no difference between the subgroups but all participants were able to complete the task with the Da Vinci X under the allotted time (27/27), whilst no participants managed to complete the task on the Versius system (0/27) [30].

### Influence of prior robotic experience with skill transfer

From repeated assessments on the Versius, Butterworth et al. reported the largest margin of improvement of GEARS amongst those with extensive robotic experience (+ 4.3) compared to those with some (+ 1.5) or no (+ 2.7) robotic experience. This was also reflected in the Versius simulator, whereby those with extensive robotic experience performed the best in the majority of tasks. This suggests prior robotic experience, despite being of a different platform, permitted better performance due to transferability of skills [20].

Transferring from MP to SP platforms, the intermediate group reported a significant reduction in GEARS (−2.9) and RACE (−5.1) scores, whilst experts and novices reported no significant change in GEARS (−2.0) and RACE (−2.0), and novices’ GEARS (+ 0.8) and RACE (+ 0.4) [29].

In Sighinolfi et al., robotic surgeons, with previous Da Vinci experience, completed tasks significantly faster than the naïve participants (*p* = 0.002) [31]. Further details on performance based on competency group can be viewed at Table 3.

**Table 3:** Subgroup definitions and performance outcomes

Author, Year ^a^	Measures of effect	Novice		Intermediate		Expert	
		Definition	Outcome	Definition	Outcome	Definition	Outcome
Butterworth et al. 2021 [20]	Performance progression of first Versius use	< 5 robot procedures	GEARS: +2.7 increase	5 to < 30 procedures	GEARS: +1.5 increase	≥ 30 robotic procedures	GEARS: +4.3 increase
Ghazi et al. 2023 [29]	Performance difference when transferring between da Vinci MP to SP	< 10 MP cases and no prior SP experience	GEARS: stable (17.3 to 18.1, −0.8) RACE: stable (15.9 to 16.3, + 0.4)	> 5 years of experience and > 250 cases of robotic MP cases and no SP experience	GEARS*: significant − 2.9 decrease (27 to 24.1) RACE*: significant − 5.1 decrease (26.9 to 21.8)	> 250 cases in MP and > 50 cases of SP	GEARS: stable (27.0 to 25.0, −2.0) RACE: stable (25.7 to 23.7, −2.0)
Reitano et al. 2023 [30]	GEARS score after one attempt on each robotic platform	Medical or non-medical students	GEARS: no difference (did not report specific scores)	-	-	-	-
Sighinolfi et al. 2023 [31]	Simulation performance scores between competency groups	No surgical experience	Task completion time*: median 60.0s (53.0–71.5.0.5)	-	-	Robotic surgeons with > 1 year of Da Vinci console experience	Task completion time*: median 38.5s (34.4–56.3)

* *p* < 0.05

^a^ Larkins et al. 2022 [21] was not included in this table as no competency groups were used in the study

### Participant perceptions

Between the Hugo RAS and the dV-trainer, 50% reported that the skills were ‘absolutely transferable’, whilst the remaining 50% reported they were ‘somewhat transferable’ [21] Between the SP and the MP system, participants gave the SP platform higher SURG-TLX scores, indicative to more workload and cognitive load, and difficulty ratings [29]. The intermediate group that had a significant decline in performance metrics also gave significantly higher SURG-TLX scores and difficulty scores when transferring to SP system.

### Risk of bias

**Fig. 2:**
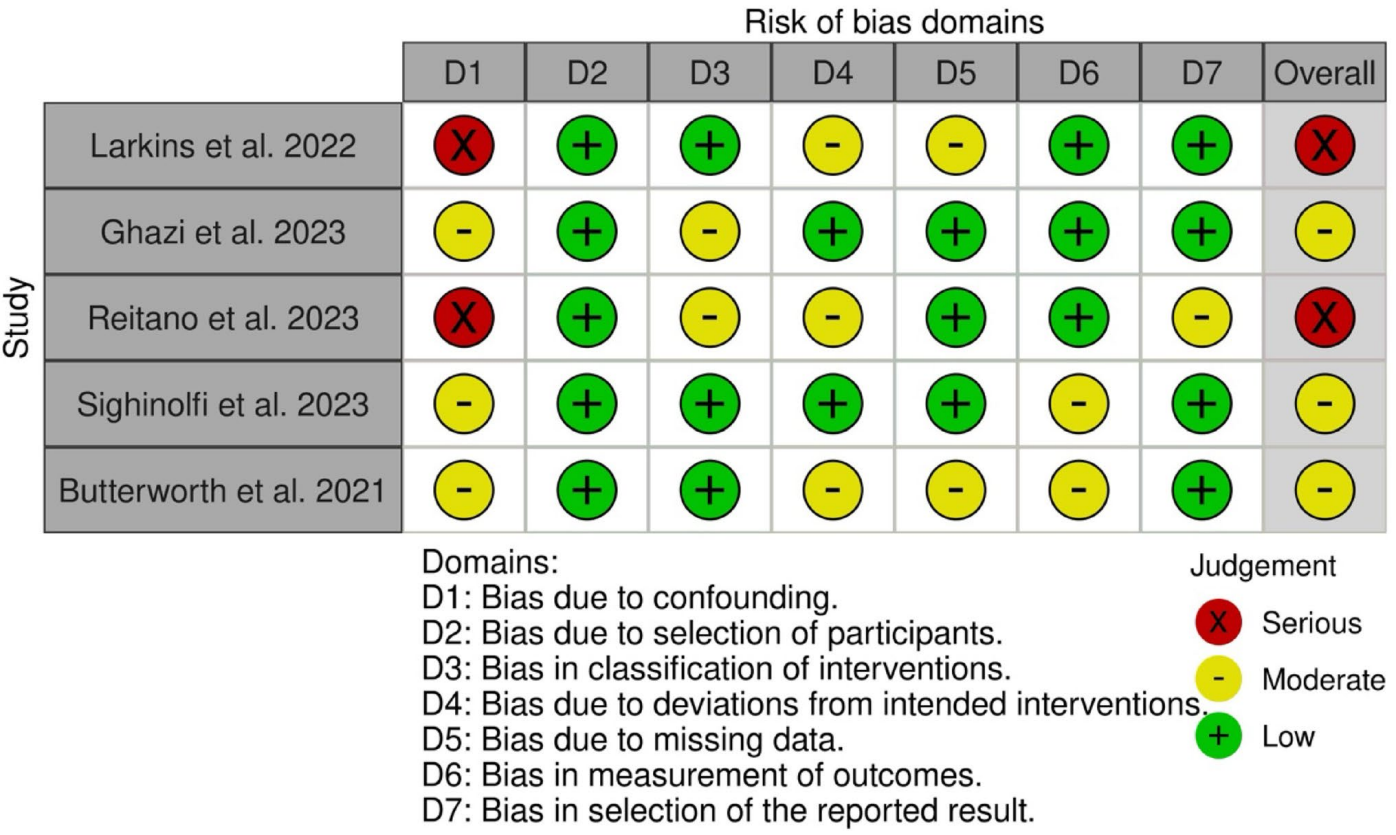
‘Traffic light plots’ of the domain-level judgements for each included study

**Fig. 3:**
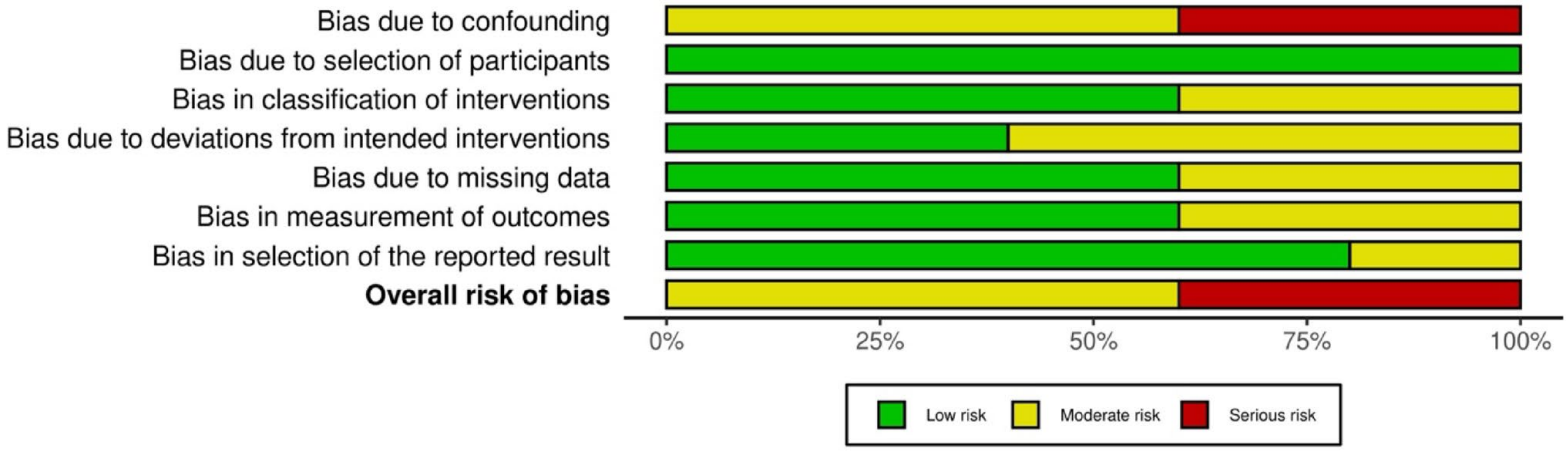
Weighted bar plots of the distribution of risk-of-bias judgement within each bias domain

Results of the risk of bias assessment can be seen in Figs. 2 and 3. Three were assessed to have moderate risk of bias [19, 29, 31] and two studies were assessed to have serious risk of bias [20, 30].

## Discussion

Whilst skill transferability between robotic platforms has been assessed with experienced robotic surgeons, it has not previously been systematically investigated in surgical trainees and in an education setting. Previous studies have focussed on outcomes from live operations, with assessment methods relying predominantly on operative timings and clinical outcomes [23, 24, 32]. However, objective evaluation in the live operating room inherently carries bias, as performance is influenced by patient complexity, intraoperative variability, and case-specific factors that frequently confound comparisons across surgeons. Thus, this present review provides novel insights to unexplored but important topic of how robotic training and skills acquisitions will vary across robotic platforms. The aim was to evaluate the effect and skill transferability of cross-platform training on robotic skills.

In spite of a comprehensive search, only five studies were found to suitably address the review’s question on the degree of skill transferability between robotic platforms. This paucity of literature is likely due to various factors including the relative novelty of multi-platform use in surgical training, the presence of multiple systems in a single training centre and the recent market entry and approval of these newer systems.

The five studies included in this review showed that there is a degree of skill transferability between robotic platforms. Although not explicitly defined in the included studies, skill transfer was observed when performance in a measured domain remained constant or improved across different robotic platforms, thus measuring immediate, cross-platform performance. This systematic review has identified that the individual’s prior robotic experience was the most prominent surgeon-related factor influencing skill transferability.

The studies in this review, which had participants with heterogeneous robotic experience, revealed varying skill transferability profiles for each expertise level. Novices and experts demonstrated consistent performances when crossing between platforms supporting skills transfer. For novices this would indicate the simultaneous development of fundamental robotic skills, which are more easily transferable across platforms. In contrast, experts consistently outperformed all other competency groups, reflecting their existing technical proficiency and ability to adapt to subtle differences in console design [29, 30]. This adaptability facilitates skill transfer between systems and obviates the need for a complete relearning process.

Importantly, in the intermediate group, variable transfer outcomes were exhibited [20, 29]. Ghazi et al. reported a lack of skill transfer in their intermediate group, whilst Butterworth et al. reported a small degree of positive skill transfer. Evidence of limited skill transfer at this stage suggests that, as surgeons develop more advanced techniques, these are not as readily transferable across systems. This has significant implications for training, highlighting the need for tailored strategies to deconstruct entrenched, system-specific habits in intermediates and thereby facilitate more effective skill transfer. Trainers, in particular, must be acutely aware of the potential risk of negative skill transfer when switching trainees between different robotic systems, and should structure curricula to minimise this risk. Understanding these distinctions would allow for further optimisation of training design.

Variation in platform design may also have a significant impact on skill transfer. Differences in console and controller configurations can affect how readily skills are acquired or transferred, with some systems appearing more user-friendly for novices [21, 30]. For example, Larkins et al. reported that one platform was perceived as more intuitive during a crossover study, although platform de-identification prevented formal attribution. Reitano et al. observed that all participants were able to complete tasks on the Da Vinci X, while none could on the Versius. Amongst intermediates, Ghazi et al. found limited transferability from the Da Vinci Xi to the Da Vinci SP system, with only certain subdomains such as depth perception, efficiency, and selected technical skills retained. These findings reflect fundamental design differences: the Versius platform places all robotic controls on the handheld units, whereas Da Vinci and Hugo RAS systems distribute controls across handhelds and foot pedals [33]. Similarly, the Da Vinci SP employs a single-port design, which can reduce triangulation, restrict lateral motion, and increase the likelihood of instrument collisions, particularly in complex tasks such as suturing [34]. Collectively, these examples illustrate how platform-specific design features can either facilitate or hinder skill transferability.

Beyond platform-specific ergonomics, task complexity is a critical, under-reported confounder when comparing skill transfer across studies. The included studies used a spectrum of tasks, including simple psychomotor stimulations using the platform’s own simulated platform, or with dry-lab models to complex, integrated procedural tasks on wet-lab models. The variable performance amongst intermediates, in particular, show they can be more vulnerable to disruption depending on the task complexity.

Successful skill transfer in live surgical setting has been reported in expert surgeons between various robotic systems, such as between the Da Vinci and the Hugo RAS, without significant loss of patient outcomes or performance [23, 24, 32]. These studies only included expert surgeons which, as shown in this review, have the ability to successfully transfer between platforms. This transferability, however, has shown to be less robust for surgeons with reduced experience. For these individuals, training on a single robotic system may not be sufficient to ensure procedural competence and patient safety when using a different system in the operating theatres. This underscores the necessity of implementing a structured, cross-platform training curriculum. Such curriculum should integrate two key components: firstly, a generalised cross-platform training exposing trainees to the nuances and ergonomic differences between platforms, and secondly, platform-specific proficiency training on the system used by their home institution. This is complemented with the initial use of VR simulations for basic skills training, then advancing to higher fidelity dry- or wet-lab models. This dual approach ensures that surgeons could effectively acquire skills for their institution’s primary platform, but also fostering adaptive expertise necessary for efficient skill acquisition on other systems.

Ultimately, a tailored training programme that accounts for prior experience and the specific characteristics of different robotic platforms is essential to address the growing diversity of systems available for surgical use. Current robotic training curricula already integrate various modalities, such as virtual reality simulators and dry-lab models, each with distinct advantages and limitations [8, 11, 35]. Incorporating simulation-based, cross-platform training would enable trainees to refine their technical skills while maintaining patient safety. This approach aligns with Cognitive Flexibility Theory, which suggests that exposure to multiple representations of similar content across differing contexts promotes deeper understanding and adaptability [36, 37]. By applying this framework to robotic surgery education, multi-platform training could strengthen understanding of fundamental robotic principles and facilitate smoother transfer of skills between systems.

Further research should explore how best to integrate cross-platform training into existing curricula. This includes comparing the effects of multi-platform versus single-platform approaches on learning curves and skill acquisition, transfer effects amongst trainees with varying experiences, and conducting longitudinal studies to characterise the trajectory of skill transfer over time. Developing and validating assessment tools and proficiency benchmarks across systems would also support a more evidence-based approach to robotic education. Importantly, such work has direct implications for training, as both surgeons and trainees should recognise the potential decline in technical performance when transitioning between robotic systems mid-training, particularly during the early stages of their learning curves.

### Strengths and limitations

Limitations to this review include the small sample sizes, low number of repetitions, heterogeneity in assessment methods and the low quality of some studies. Although the assessment methods have previously been validated [38, 39], heterogeneity resulted to lack of meta-analysis. Furthermore, each study had their own definition of ‘novice’, ‘intermediate’ and ‘expert’, whereby the criteria was based on years, or caseload or both. On the other hand, the strengths of this study included a comprehensive search strategy through multiple databases, robust blind screening and analysis of data between two reviewers, thorough quality assessment of the studies and a novel systematic review on this topic.

## Conclusion

Variation in skill transferability was observed across robotic surgical platforms, influenced by the level of surgical experience. Novices and experts demonstrated relatively uniform learning patterns across systems, whereas trainees with some prior robotic exposure exhibited more restricted transfer, highlighting the need for targeted training to address specific skills that do not readily translate between platforms. Prior robotic experience appears to play a major role, with greater experience associated with improved performance and faster progression. However, heterogeneity in study design and methodological limitations constrain definitive conclusions. Further research is required to clarify the extent to which cross-platform training can be systematically integrated to optimise robotic surgical education. This can be addressed with the use of prospective randomised controlled trials to isolate the causal effects of such curricula and define evidence-based, platform-agnostic proficiency benchmarks for credentialing.

## Supplementary Information

Below is the link to the electronic supplementary material.

Supplementary Material 1

## Data Availability

No datasets were generated or analysed during the current study.
