# Supplementary material for: Assessing effectiveness and skill transferability in multi-platform simulated training for robotic surgical skills: a systematic review
Source: J Robot Surg. 2025 Dec 29;20(1):121. doi: 10.1007/s11701-025-03090-x (PMC12748306; doi:10.1007/s11701-025-03090-x)
Supplement: Supplementary file 1 — Supplementary Material 1 [file 11701_2025_3090_MOESM1_ESM.pdf]

Supplementary Information: Search Terms and Strategy

Supplementary information A. Search Terms

(“simulat*” OR “robot*” OR “virtual simulation*” OR “dry lab” OR “cadaveric” OR “robot* train*”)

AND

((Da Vinci* OR Davinci*) AND (Hugo* OR Versius* OR Hinotori* OR Kang* OR Revo* OR Senhance*))

OR (Hugo* AND (Versius* OR Hinotori* OR Kang* OR Revo* OR Senhance*))

OR (Versius* AND (Hinotori* OR Kang* OR Revo* OR Senhance*))

OR (Hinotori* AND (Kang* OR Revo* OR Senhance*))

OR (Kang* AND (Revo* OR Senhance*))

OR (Revo* AND Senhance*)

Supplementary information B. Search Strategy

Table B1. Search strategy in PubMed, search conducted on 20 January 2025

Query	Results
(“simulat*” OR “robot*” OR “virtual simulation*” OR “dry lab” OR “cadaveric” OR “robot* train*”) AND ((Da Vinci* OR Davinci*) AND (Hugo* OR Versius* OR Hinotori* OR Kang* OR Revo* OR Senhance*)) OR (Hugo* AND (Versius* OR Hinotori* OR Kang* OR Revo* OR Senhance*)) OR (Versius* AND (Hinotori* OR Kang* OR Revo* OR Senhance*)) OR (Hinotori* AND (Kang* OR Revo* OR Senhance*)) OR (Kang* AND (Revo* OR Senhance*)) OR (Revo* AND Senhance*)	306

Table B2. Search strategy in Medline, search conducted on 20 January 2025

Query	Results
(“simulat*” OR “robot*” OR “virtual simulation*” OR “dry lab” OR “cadaveric” OR “robot* train*”) AND ((Da Vinci* OR Davinci*) AND (Hugo* OR Versius* OR Hinotori* OR Kang* OR Revo* OR Senhance*)) OR (Hugo* AND (Versius* OR Hinotori* OR Kang* OR Revo* OR Senhance*)) OR (Versius* AND (Hinotori* OR Kang* OR Revo* OR Senhance*)) OR (Hinotori* AND (Kang* OR Revo* OR Senhance*)) OR (Kang* AND (Revo* OR Senhance*)) OR (Revo* AND Senhance*)	196

Table B3. Search strategy in Embase, search conducted on 20 January 2025

Query	Results
(“simulat*” OR “robot*” OR “virtual simulation*” OR “dry lab” OR “cadaveric” OR “robot* train*”) AND ((Da Vinci* OR Davinci*) AND (Hugo* OR Versius* OR Hinotori* OR Kang* OR Revo* OR Senhance*)) OR (Hugo* AND (Versius* OR Hinotori* OR Kang* OR Revo* OR Senhance*)) OR (Versius* AND (Hinotori* OR Kang* OR Revo* OR Senhance*)) OR (Hinotori* AND (Kang* OR Revo* OR Senhance*)) OR (Kang* AND (Revo* OR Senhance*)) OR (Revo* AND Senhance*)	401

Table B4. Search strategy in Scopus, search conducted on 20 January 2025

Query	Results
(“simulat*” OR “robot*” OR “virtual simulation*” OR “dry lab” OR “cadaveric” OR “robot* train*”) AND ((Da Vinci* OR Davinci*) AND (Hugo* OR Versius* OR Hinotori* OR Kang* OR Revo* OR Senhance*)) OR (Hugo* AND (Versius* OR Hinotori* OR Kang* OR Revo* OR Senhance*)) OR (Versius* AND (Hinotori* OR Kang* OR Revo* OR Senhance*)) OR (Hinotori* AND (Kang* OR Revo* OR Senhance*)) OR (Kang* AND (Revo* OR Senhance*)) OR (Revo* AND Senhance*)	331

Table B5. Search strategy in Clinicaltrials.gov, search conducted on 20 January 2025

Query	Results
(“simulat*” OR “robot*” OR “virtual simulation*” OR “dry lab” OR “cadaveric” OR “robot* train*”) AND ((Da Vinci* OR Davinci*) AND (Hugo* OR Versius* OR Hinotori* OR Kang* OR Revo* OR Senhance*)) OR (Hugo* AND (Versius* OR Hinotori* OR Kang* OR Revo* OR Senhance*)) OR (Versius* AND (Hinotori* OR Kang* OR Revo* OR Senhance*)) OR (Hinotori* AND (Kang* OR Revo* OR Senhance*)) OR (Kang* AND (Revo* OR Senhance*)) OR (Revo* AND Senhance*)	0
